# Correction to: Niche differentiation among facultative filter feeders: Insights from invasive Ponto-Caspian mysids

**DOI:** 10.1093/cz/zoad044

**Published:** 2023-10-07

This is a correction to: Péter Borza, Varsha Rani, Csaba F Vad, Niche differentiation among facultative filter feeders: Insights from invasive Ponto-Caspian mysids, Current Zoology, 2023;, zoad030, https://doi.org/10.1093/cz/zoad030

In the originally published version of this manuscript, incorrect funding project’s ID number was inadvertently included in the Acknowledgements section.

The affected sentence has been corrected to read as follows:

“VR acknowledges further support by the NKFIH-138215 project.”

